# The Influence of the Information Richness of Interfaces on Consumers’ Purchase Intention: The Sequential Mediating Effects of Cognitive Load, Mental Imagery, and Flow Experience

**DOI:** 10.3390/bs15050673

**Published:** 2025-05-14

**Authors:** Jiayue Guo, Yuemeng Zhao, Wenqian Zhang, Ke Lu, Xiaochen Feng, Tiansheng Xia

**Affiliations:** 1 School of Art & Design, Guangdong University of Technology, Guangzhou 510090, China; guojiayue@gdut.edu.cn (J.G.); 2112217078@mail2.gdut.edu.cn (Y.Z.); 2112417036@mail2.gdut.edu.cn (W.Z.); 2112317066@mail2.gdut.edu.cn (K.L.); 2 School of Public Administration and Policy, Dalian University of Technology, Dalian 116024, China; 2112417073@mail2.gdut.edu.cn

**Keywords:** e-commerce live streaming, interface design, flow, cognitive load, purchase behavior

## Abstract

E-commerce live streaming attracts consumers by displaying product information and anchor introductions. However, the complexity and variety of interface information pose challenges in design, and research on live-streaming interface design remains limited. This study examines how patch design affects the customer experience in live-streaming rooms, considering different product types and virtual backgrounds. Based on the flow theory and the Stimulus–Organism–Response model, we conducted experiments simulating live-streaming scenarios and collected behavioral and eye-tracking data. Our results confirmed that cognitive load negatively mediates, while mental imagery and flow experience positively mediate, the relationship between patch design and consumer behavior. Additionally, the interaction between product type and virtual background proximity was revealed, with product type moderating the effect of cognitive load on purchase behavior. This study contributes to understanding the impact of live-streaming interface design on consumer experience and purchase behavior, providing design guidelines for online retailers and managerial insights for retail platforms.

## 1. Introduction

With the rapid development of communication technology, mobile browsing is becoming increasingly popular, and web browsing is losing popularity (Perakakis et al., 2015), especially e-commerce live streaming, which has become popular on mobile platforms used by the traditional e-commerce, content creation, and shopping guide communities in China. The rise of live-streaming commerce has revolutionized online shopping, shifting consumer behavior from static “web-browsing” to dynamic “mobile interaction” (M. Li et al., 2022; Liao et al., 2016). While this shift enhances user engagement through real-time visuals, mobile devices’ limited screen size imposes critical constraints: dense information presentation risks, cognitive overload, and poor interface designs reduce satisfaction (Ghose et al., 2013; Sohn et al., 2017). The small interface of cell phones hinders information perception (Ghose et al., 2013), and the visual complexity of this interface may pose significant challenges to mobile online store users (Sohn et al., 2017). To mitigate these challenges, platforms increasingly adopt “patches”—scrollable banners displaying product details via text, images, or hybrid formats. However, their design efficacy remains under-tested, particularly regarding how different formats balance information richness and cognitive demand (Groß, 2015; Kang et al., 2021). Visual element design and interface design are key components of environmental stimuli in research on online consumer behavior. Specifically, visual element design has been studied in relation to task-related cues (Ha & Lennon, 2010), interactive text in chat and comment sections (Fei et al., 2021), and other visual cues. Interface design, on the other hand, generally focuses on broader aspects of user interaction and navigation. (Fei et al., 2021). In the existing research, there are few disaggregation studies that focus on the information richness of the interface elements, i.e., patches.

In this context, the Stimulus–Organism–Response (S-O-R) theory offers a valuable framework for understanding the dynamics at play. Initially proposed by Skinner in 1935, the theory posits that the environment influences human behavior by shaping responses to stimuli (Mehrabian & Russell, 1974). Mehrabian later expanded on this by identifying three key components, i.e., stimulus, organism, and response, which correspond to antecedent variables, mediating variables, and outcome variables, respectively (L. Liu et al., 2025). In online shopping environments, the S-O-R model is particularly relevant, as it illustrates how environmental stimuli—such as interface and product presentation—affect consumer sentiment, which then drives behavioral responses, like purchase behavior. Several studies have applied the S-O-R framework to show that interface design acts as an antecedent, shaping emotional responses and mediating the relationship between online store interfaces and consumer purchasing decisions (Wu et al., 2008).

Visual elements and interface design are key components of environmental stimuli in online consumer behavior. In live-streaming commerce, cognitive load is an important “organism” factor, referring to the mental effort required to process information. According to the cognitive load theory, there are three types of cognitive loads: intrinsic load, extraneous load, and germane load. Intrinsic load is related to the inherent difficulty of comprehending the material, extraneous load pertains to how information is presented, and germane load involves the effort invested in integrating new information with existing knowledge (Leppink et al., 2013). Studies have shown that small screen sizes increase cognitive load by making tasks like typing and searching more difficult, which negatively impacts task performance (Faudzi et al., 2024). It has been shown that screens negatively affect task performance by increasing cognitive load and making tasks such as typing and searching more difficult. Therefore, it is very important to investigate the cognitive state of consumers in mobile live shopping.

The unique characteristic of live streaming is that consumers can interact with sellers in real time, resulting in an immersive, engaging shopping experience and a more interpersonal connection (Wongkitrungrueng & Assarut, 2020). This experience is also known as the flow experience. The flow theory, a cornerstone in online consumer behavior research, posits that immersive experiences (e.g., control, interest, etc.) mediate between environmental stimuli and behavioral responses. Prior studies have focused on broad factors like platform utility, entertainment, and social presence (van Noort et al., 2012; L. Wang & Wang, 2020), yet have neglected mobile-specific design elements.

A key aspect of how consumers engage with online content is through psychological imagery, often referred to as “visualization” or “seeing with the mind’s eye”. This process involves representing non-verbal information in working memory. Visual stimuli, such as vivid images, play a significant role in generating mental imagery, which in turn enhances consumer engagement and decision making (Adaval et al., 2019; Gavilan et al., 2020). With the rise in interactive experiences in online shopping, such as digital gaming and virtual models, technology has further stimulated imagery, amplifying consumer involvement in decision-making processes (Hu et al., 2017).

By capturing infrared reflections from the pupil through image processing principles, eye-tracking technology records eye movements using special cameras to track eye motion (H.-C. Chen et al., 2022). Eye tracking, owing to its scientific rigor and ease of use, has become a preferred research method in various fields, including live-streaming, cognitive load, and flow experience studies (Klaib et al., 2021). Through the eye-tracking technology, in the context of live shopping, we can understand how patches of different information richness affect consumers’ attention and decision making and gain a deeper understanding of how cognitive load is managed and how information presentation affects their purchasing behavior.

This research investigates a framework for understanding how interface design influences customer retention and purchase decisions in the context of live-streaming platforms. This study collects behavioral data and eye-tracking data through a mixed research approach, providing an in-depth analysis of the mechanisms affecting customer experience on live-streaming platforms. The findings contribute to a deeper understanding of consumer behavior in the rapidly expanding field of live-streaming commerce.

## 2. Literature Review and Research Model

### 2.1. The Effect of Information Richness on Purchase Intention

Information richness affects purchase intention, web interface design significantly affects consumer behavior, and a high-quality user interface can improve purchase intention (Cho et al., 2023). This conclusion is consistent with that drawn by (Patel et al., 2020), who found a positive correlation between the quality of mobile shopping apps and consumers’ purchase intentions, indicating that an enhanced app design has a positive impact on shopping behavior through improved functionality and user engagement. Studies have also shown that an effective design can alleviate concerns, thereby improving consumer satisfaction and loyalty, and thus driving purchase intention (Kng & Chang, 2022). The patch of the broadcast room interface is designed to enrich the interface information. The information richness provided by the patch in the screen size of the mobile phone can be used as an external cue to reduce the cognitive load in the purchase decision process. This convenience can increase consumer satisfaction and enhance purchasing behavior in the online environment. Therefore, the following hypothesis is proposed:

**H1.**  *The information richness of the patch design of the live-streaming interface has a significant positive effect on consumers’ purchase intention.*

### 2.2. Information Richness, Mental Imagery, and Cognitive Load

The theory of flow experience has become one of the main theories to explore consumers’ online shopping behavior. Flow experience is an important concept in the online business environment and a source of value for marketers (van Noort et al., 2012) and is an influential variable in consumer engagement research (Csikszentmihalyi, 2020), including the dimensions of control, interest, attention, and curiosity (Tsai & Pai, 2012; L. C. Wang et al., 2007). This is an intense, optimal state of consciousness resulting from a high level of focus on a task (Cseh et al., 2015), at which point users typically find themselves disconnected from the real world and immersed in an activity that they feel is a natural and enjoyable experience or a spiritual experience (Brannon Barhorst et al., 2021; Schouten et al., 2007).

According to the dual coding theory (Paivio, 1990), multimodal presentations require simultaneous verbal and visual processing, generating a higher cognitive load than unimodal formats. The deployment of cognitive resources correlates with presence perception (Sas & O’Hare, 2003), suggesting that richer information designs (e.g., integrated graphics–text) may better stimulate flow through enhanced engagement (Guan et al., 2022; Sun et al., 2019). This cognitive load continuum aligns with flow theory’s challenge–skill balance principle, where integrated designs provide the optimal challenge for flow induction (Keller et al., 2011), while text-only designs may fail to initiate necessary cognitive engagement.

Mental imagery, often referred to as “visualizing” or “seeing with the mind’s eye”, is the process of representing non-verbal information in working memory. Rich, vivid visual cues in online shopping interfaces, such as those found in combined text and pictures, enhance the generation of mental imagery and contribute to greater consumer engagement and decision making (Jia et al., 2021). It has been argued that information-rich patches, especially those combining text and graphics, lead to richer mental imagery and a more immersive flow experience for consumers. A According to the construal level theory, abstract information presented at a higher level can stimulate more vivid mental imagery. This has been supported by empirical evidence showing that visually stimulating content enhances the generation of mental images and promotes greater consumer engagemen (Jia et al., 2021; Yan et al., 2016). While pictures can sometimes disrupt the integration of verbal information, leading to cognitive overload (Adaval et al., 2007), the combination of pictures and text can also enhance engagement when appropriately balanced (Xiang et al., 2016). Research has shown that multimedia presentations can sometimes lead to cognitive overload when they are too complex, especially in environments like online shopping (Chang et al., 2015; Sohn et al., 2017). However, when combined in a complementary manner, such as in advertising or educational contexts, visual and textual elements can reinforce each other and improve outcomes (Ildirar et al., 2018).

Cognitive load refers to the total amount of mental effort being used in working memory and has significant implications for various experiences, including the flow state—a highly focused mental state characterized by immersion and enjoyment in activities (Zhang et al., 2014). Flow is positively associated with consumers’ intention to stay on the website for a longer period of time (Kabadayi & Gupta, 2005) and determines the success of a website (Hsu et al., 2013). It is well documented that an increase in cognitive load can adversely affect the flow experience. This occurs as cognitive overload hinders the individual’s ability to remain fully engaged and immersed in a task, leading to diminished performance and enjoyment. When cognitive resources are overtaxed, the quality of attention shifts from the task at hand to managing stressor-related cognitions, which further derails the flow experience (Leppink et al., 2013). Therefore, the following hypotheses are proposed:

**H2a.**  *The information richness of patch design affects cognitive load, with text-only designs leading to higher levels of cognitive load and graphic–text combinations leading to lower levels of cognitive load.*

**H2b.**  *The information richness of patch design affects mental imagery, with the lower-level imagery evoked by text-only designs and the higher-level imagery evoked by graphic combinations.*

**H2c.**  *The information richness of patch design positively affects flow, with the lower level flow induced by text-only designs and the higher level flow induced by graphic–text combinations.*

An increased cognitive load is closely linked to distraction. When individuals are faced with high-load tasks, their attention is often focused on the basic elements of the task, resulting in the neglect of other important information (Leppink et al., 2013). This phenomenon, known as “attentional blindness”, is particularly evident in complex decision-making or clinical situations. Studies show that, in such contexts, individuals, such as clinicians, may miss important visual cues due to a high cognitive load. Moreover, an excessive cognitive load hinders the generation of mental imagery (Mohan et al., 2014). In the shopping context, consumers often face indecision or “buying entanglement”, which can occur when product information is not sufficiently rich or clear. A high cognitive load inhibits mental imagery, and when richer images are provided, psychologically proximal products can prevent users from effectively processing these images, leading to lower product ratings (Jia et al., 2021). Therefore, an excessive mental load impedes information processing and can result in a poor user experience. Based on this understanding, we hypothesize the following:

**H3a.**  *Cognitive load negatively affects flow.*

**H3b.**  *Cognitive load negatively affects mental imagery.*

Flow in live e-commerce primarily occurs during entertainment-focused activities such as live streaming (Novak et al., 2003; van Noort et al., 2012), where users’ attention is focused on the product presentation, which requires minimal cognitive effort compared to more complex actions. In contrast, mental imagery plays a significant role in altering emotional states, both positive and negative (Pictet & Holmes, 2013). It triggers thoughts and emotional responses by simulating non-current events through sensory representations, and in psychological contexts, pictures act as “emotional amplifiers” for a wide range of emotions (Holmes et al., 2008). In live streaming, entertainment positively influences viewers’ flow experience (C.-C. Chen & Lin, 2018; Y. Li & Peng, 2021). The visual appeal and engaging nature of live broadcasts, such as in travel broadcasts, enhance the pleasure, joy, and happiness of viewers (Eroglu et al., 2001; van der Heijden et al., 2003). Given that live-streaming interfaces are dominated by the host, creating a pleasing atmosphere, mental imagery may serve as an amplifier of positive emotions, which, in turn, facilitates flow. Therefore, the following hypothesis is proposed:

**H4.**  *Mental imagery positively influences the experience of flow.*

The cognitive load typically affects customers’ emotions, decision-making processes, and purchase behavior. When their cognitive load exceeds an individual’s processing capacity, it can result in decreased decision quality, increased emotions like stress or anxiety, and impaired judgments (Mohan et al., 2014). In AR shopping, excessive choice from a high cognitive load creates confusion, dissatisfaction, and uncertainty, which reduces willingness to buy, while lower cognitive load enhances purchase intention and willingness to pay (Barta et al., 2023). In live shopping, the cognitive load generated by the patch design may have a similar effect, as the cyclic scrolling of products in the scroll bar gives customers too many choices and creates uncertainty in the purchase decision. It has also been shown that a high cognitive load diminishes the effect of imagery, and mental imagery promotes customers’ evaluation of products (Jia et al., 2017, 2021); thus, mental imagery may favor customers’ willingness to purchase. There is substantial research confirming the role of flow in enhancing website experience, purchase behavior (Landers et al., 2015), and satisfaction (Hsu et al., 2013). For instance, Waterlander et al. demonstrated that engagement levels in virtual environments, like a virtual supermarket, directly influenced purchase decisions. This suggests that flow experiences, generated through immersive environments, can significantly enhance real-life purchase behavior and decision making (Waterlander et al., 2015). Therefore, the following hypotheses are proposed:

**H5a.**  *Cognitive load negatively affects purchase intentions.*

**H5b.**  *Mental imagery positively influences purchase intentions.*

**H5c.**  *Flow positively influences purchase intentions.*

### 2.3. The Effect of Product Type on Purchase Intention

Search products are products whose quality can be judged by consumers through searching for information before purchase, such as electronic products (Nelson, 1974). Consumers do not need to try or experience these products before purchasing them; instead, they determine the quality based on the main parameters that are available via an information search. Contrarily, experience goods are products whose quality cannot be determined before purchase, such as clothing, milk, and perfume. For these products, consumers often rely on sensory experiences and personal feelings to judge their quality, making it more challenging to evaluate them through just information. The cognitive load required to evaluate these products differs based on the type. Search goods generally require extensive information search and comparison, leading to a higher cognitive load. Experience goods require more emotional and sensory evaluations, often leading to a lower cognitive load. However, when the patch information is not clear or accessible in online shopping, cognitive load increases, which can negatively impact purchase decisions (Klein & Melnyk, 2016). In live shopping scenarios, search products often require an extensive information search, leading to a higher cognitive load due to the need for comparison and evaluation. In contrast, experience products rely more on emotional and sensory engagement, which may result in different purchase intentions and behavior patterns, especially in a live-streaming context (Voss et al., 2003). Therefore, we propose the following hypothesis:

**H6.**  *The moderating effect of product type on cognitive load and its influence on purchase intentions.*

**Figure 1:**
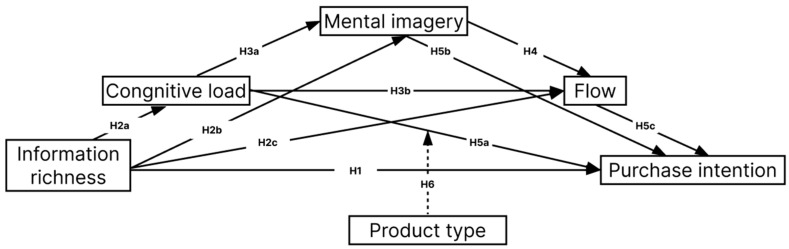
Research model.

Based on the above reasoning, we integrate flow experience, cognitive load, and psychological imagery into the S-O-R framework (see Figure 1). This model allows us to capture the multiple layers of interaction between design elements and consumer behavior, offering a comprehensive understanding of how information richness influences purchasing decisions in live-streaming commerce.

## 3. Materials and Methods

### 3.1. Participants

A total of 164 participants, aged between 18 and 24 years (M = 21; SD = 1.8), were recruited via an online platform at Guangdong University of Technology. All participants provided informed consent before participating in the experiment. This study was approved by the Ethics Committee of Guangdong University of Technology. Participants were randomly assigned to two groups (functional products and experience products), and each group completed the experiment under three different experimental conditions.

### 3.2. Experimental Design

In this study, we aimed to combine physiological and behavioral experiments to explore the psychological mechanisms behind live streaming and their effects on purchase intention. The experiment employed a 3 (patch design information richness: text, image, and graphic–text combination) × 2 (product type: search product vs. experience products) design. Each group was exposed to the three conditions based on patch design information richness. The primary focus of this experiment was not to examine interaction effects between product type and patch design, but rather to investigate the moderating role of product type on cognitive load. Specifically, we hypothesize that cognitive load may differ between the search and experience product groups, with the goal of testing this moderating effect on consumer behavior, including cognitive load, flow, mental imagery, and purchase intention.

#### 3.2.1. Experimental Equipment and Stimulation Material

To examine how the live shopping situation facilitates or hinders the customer experience, this study used the interface design software Figma (https://www.figma.com/), the video-editing software Adobe Premier (https://www.adobe.com), and the FlashCut App (https://www.flashcut.cn/) to create a live video to simulate a real scenario and used the Experiment Builder to write an eye-tracking program, in order to obtain physiological data.

Considering the generalizability of this study, a more representative hedonic product (perfume) and functional products (monitor) were chosen as experimental materials. Experience products are products that are evaluated after purchase or use, such as beauty and makeup products, while hedonic products can be evaluated by parameters, such as computing devices (Kim, 2019; Sheng et al., 2021). In this study, the manipulation of information richness in patch design was achieved by varying the content presented to the participants. Specifically, we created three levels of patch design richness: text, picture, and graphic–text combination. The text condition contained only textual information, the image condition featured only visual elements (pictures), and the graphic–text combination condition combined both textual and visual information. These variations were designed to reflect different levels of information richness and explore their impact on cognitive load, flow, and purchase intention.

**Figure 2:**
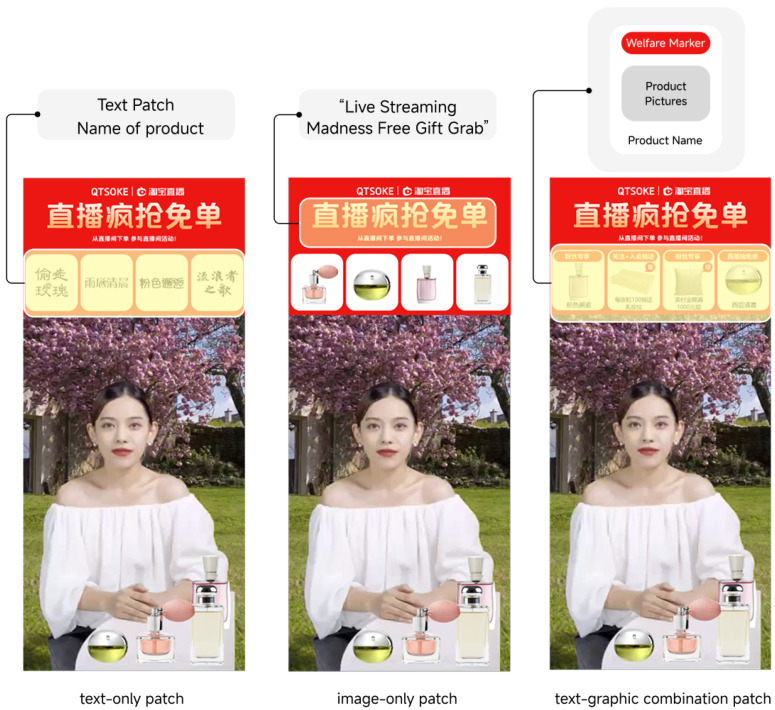
One example of experimental materials for the live-streaming interface.

Figure 2 shows the three experimental materials used to manipulate information richness: a text-only patch, a picture-only patch, and graphic–text combination patch. To assess the effectiveness of the experimental materials, we invited 31 additional participants to evaluate three images (shown in Figure 2) before the formal experiment. Participants rated the images based on the following dimensions using a 5-point Likert scale (1 = strongly disagree, 5 = strongly agree): information richness (M = 3.6, SD = 0.915), clarity (M = 3.68, SD = 0.979), complexity (M = 2.00, SD = 0.817), and relevance to the live shopping environment (M = 3.14, SD = 0.807). These evaluations suggest that the materials were perceived as sufficiently rich and clear, with moderate complexity, and were considered reasonably representative of the live shopping environment.

#### 3.2.2. Experimental Procedures

Participants were randomly assigned to two experimental situations. The experimental procedure consisted of three steps. First, the researcher invited the subjects to come to the laboratory, explained the purpose and procedure of the experiment, had the subjects sit in a comfortable position, and obtained a consent form for this study. Then, the subjects were asked to place their heads on a support stand and begin the experiment after monocular calibration using the Eyelink 1000 Plus eye tracker (SR Research, Ottawa, ON, Canada), while another computer was used to record facial emotional responses. Participants were randomly assigned to two groups, and they watched three live videos with different patch designs in a randomized order and finally filled out a questionnaire. The entire experiment lasted approximately 20 min.

### 3.3. Measurement Methods

In this study, we used various established scales to measure key indicators related to the flow experience, cognitive load, mental imagery, and purchase behavior in a live-streaming context. Specifically, we employed scales adapted from previous research to assess the dimensions of flow, cognitive load, and mental imagery.

To measure flow in the behavioral data, we employed a standard adapted from previous research (Petter et al., 2007; Siekpe, 2005). This questionnaire assessed three dimensions of flow: perceived enjoyment, perceived control, and concentration. These dimensions were evaluated based on participants’ responses during the live-streaming experience. Cognitive load was derived from the adapted scale (Jia et al., 2021; Schmeck et al., 2015), which was used to report cognitive load while watching the live stream; the eight items of mental imagery were adapted by Sheng et al. (2021) and the purchase behavior scale (H. Liu et al., 2016) measured the willingness to purchase a product triggered by the flow.

Previous studies on flow experience have typically distributed questionnaires on websites or asked subjects to respond to survey questions after recalling their online experiences (Bao & Zhu, 2023; D. Li & Browne, 2006). Dimensions such as perceived enjoyment and perceived control are commonly used as reflective indicators of flow (Petter et al., 2007; Siekpe, 2005; L. C. Wang et al., 2007; Zhou et al., 2010). In online shopping scenarios, perceived pleasure, perceived control, and concentration have been most frequently used in empirical studies of flow (Guo & Poole, 2009; Koufaris, 2002; D. Li & Browne, 2006; L. Wang & Wang, 2020), and in many cases, they have been used to conceptualize the flow. Koufaris et al. developed a scale that seems to be most applicable to online shopping environments (Hausman & Siekpe, 2009), and therefore, the scales corresponding to these three indicators were followed in this study. However, in our measurement, some flow-related items—specifically those related to confusion, calmness, and frustration while watching the live stream—did not meet the required factor loading threshold (less than 0.3). This suggests that these items were not effective in capturing flow experiences in the live-streaming context. Consequently, these items were excluded from the final analysis, and the remaining items were used to assess flow.

Previous research has demonstrated that eye tracking is an effective method for measuring cognitive load by tracking attention distribution and fixation duration, as discussed by Rosch and Vogel-Walcutt (2013), who highlighted its role in enhancing real-time training by providing insights into attentional processes. Different presentation formats—text, image, and graphic–text combinations—were manipulated to assess their impact on cognitive load, which can be effectively measured using eye-tracking data. Flow can be measured using dwell time, as longer dwell times often indicate sustained attention and engagement, which are key characteristics of the flow state. In some cases, prolonged dwell time may also be associated with motivation and top-down attention, as goal-driven participants tend to avoid looking at irrelevant stimuli in the context (Harris et al., 2017; Mohanty & Sussman, 2013). Therefore, the relationship between dwell time and flow may reflect how participants actively engage with relevant content while filtering out distractions. Considering that subjective reports of cognitive load can be biased or imprecise, we combined eye-tracking indicators with self-report measures in the later statistical analysis to improve construct validity. Eye movement data provide a more objective complement to questionnaire-based assessments, particularly for cognitive constructs such as load and engagement.

#### Reliability and Validity Tests

The scales for cognitive load, mental imagery, flow, and purchase intention were first tested for internal consistency reliability using Cronbach’s alpha. The reliability coefficients for each scale ranged from 0.81 to 0.95, with an overall reliability of 0.90, indicating good internal consistency.

## 4. Results

After the data were collected, a small number of non-compliant samples were removed (including those with no changes in answers, inattentively answered, or incomplete responses). The final valid sample consisted of a higher proportion of women with experience in watching live shopping, with 82% of the sample being female and 18% being male.

### 4.1. Behavioral Data Analysis

We conducted a descriptive statistical analysis, and the results are shown in Table 1 which presents the means and scales for each variable under different conditions. In addition, purchase intention was significantly lower for text patches (M_text_ = 2.846) than for picture patches (M_picture_ = 3.395) and for picture patches than for graphic–text combination patches (M_graphic-text combination_ = 3.962). Fragrance products triggered significantly higher mental imagery and flow experiences than functional products: M_fragrance_ = 4.512 > M_display_ = 4.127, SD = 0.125, *p* = 0.002 and M_fragrance_ = 4.195 > M_display_ = 3.767, SD = 0.102, *p* < 0.001.

Linear mixed-effects models were conducted using the lmer Test package in R to examine the effect of patch design information richness (IR: text, picture, graphic–text combination) on purchase intention (PI), flow, cognitive load (CL), and mental imagery (MI) (Hayes, 2013).

The results indicated the following: Purchase intention (PI) was IR_Picture_ vs. IR_Text_, t(250) = 4.66, *p* < 0.001; IR_Graphic–text combination_ vs. IR_Text_, t(250) = 11.95, *p* < 0.001, *η*_p_^2^ = 0.37. Flow was IR_Picture_ vs. IR_Text_, t(250) = 3.24, *p* < 0.01; IR_Graphic–text combination_ vs. IR1, t(250) = 7.12, *p* < 0.001, *η*_p_^2^ = 0.17. Cognitive load (CL) was IR_Picture_ vs. IR_Text_, t(250) = 2.27, *p* < 0.05; IR_Graphic–text combination_ vs. IR_Text_, t(250) = 2.70, *p* < 0.01; *η*_p_^2^ = 0.24. Mental imagery (MI) was IR_Picture_ vs. IR_Text_, t(250) = 3.42, *p* < 0.001; IR3 vs. IR_Text_, t(250) = 5.47, *p* < 0.001; *η*_p_^2^ = 0.11. These findings indicate that the effects of patch design information richness on all outcome variables were statistically significant and met conventional thresholds for effect sizes.

### 4.2. Eye-Tracking Data Analysis

Statistical analyses of the eye movement data were performed using the Shapiro–Wilk normality test (Zimmerman, 2003), and a nonparametric Kruskal–Wallis test was performed for conditions in which the results did not satisfy normally distributed data in order to examine the three different patches of information for first fixation duration differences. The *p*-values in Kruskal–Wallis’s test results were lower than 0.05, indicating that the main effect of patch information was significant in the two conditions of the virtual background area of interest, the perfume distant view (*p* = 0.033) and the near-monitor view (*p* = 0.009), and the three formats of text, picture, and text and picture. There was a significant difference in the first fixation duration metric between the conditions, with means required to be added. The ANOVA test was applied to the remaining data that met the normal distribution.

The eye-tracking value of the live patch and the background were divided by their areas to obtain fixation count data per unit area for both areas of interest, and significant main effects were found for both areas of interest, and the fixation counts for patch, F(1,792) = 17.04, *p* < 0.001, *η*_p_^2^ = 0.022, first fixation time, F(1,792) = 6.54, *p* = 0.011, *η*_p_^2^ = −0.008, dwell time, F(1,792) = 300.01, *p* < 0.001, *η*_p_^2^ = 0.280, and max fix pupil size, F(1,792) = 105.917, *p* < 0.001, *η*_p_^2^ = 0.121, were all significantly smaller than the fixation count of the film (0.121), which was significantly smaller than background.

There was a significant main effect of product type, and the fixation count, F(1,792) = 5.31, *p* = 0.000, *η*_p_^2^ = 0.019, dwell time, F(1,792) = 55.26, *p* = 0.000, *η*_p_^2^ = 0.067, and max fix pupil size, F(1,792) = 5.977, *p* = 5.977, *p* = 0.015, η_p_^2^ = 0.008, were significantly higher than those of the functional products. The main effect of patch design on fixation count was significant (F (2,792) = 5.39, *p* = 0.005, *η*_p_^2^ = 0.014). The interaction effect of area of interest with product type on the unit fixation count was significant (F (1,770) = 5.73, *p* = 0.017, *η*_p_^2^ = 0.007). A further simple effect, F (1,770) = 20.75, *p* < 0.001, *η_p_*^2^ = 0.026, showed that perfume products (M_perfume_ = 59.31, SD = 2.71) had a significantly larger unit fixation count than functional products (M_display_ = 46.41, SD = 2.78) in the virtual background zone of interest. The interaction effect of region of interest with product type on the dwell time was significant (F (1,770) = 48.46, *p* < 0.001, *η*_p_^2^ = 0.059). In a further simple effects analysis of fragrance, F (1,770) = 302.50, *p* < 0.001, *η*_p_^2^ = 0.282, the dwell time was significantly greater in the virtual background area of interest (M_virtual background_ = 53,972.93, SD = 1293.99) than in the patch area of interest (M_patch_ = 22,105.04, SD = 1297.25). In a simple effects analysis of the displays, F (1,770) = 52.331, *p* < 0.001, *η_p_*^2^ = 0.064, the dwell time was significantly greater in the virtual background area of interest (M_virtual background_ = 35,080.59, SD = 1327.28) than in the patch area of interest (M_patch_ = 21,484.60, SD = 1330.66). Furthermore, there was a significant main effect of product type in the virtual background area of interest, F (1,770) = 103.87, *p* < 0.001, *η_p_*^2^ = 0.119, with a significantly greater dwell time for perfume products (M_perfume_= 53,972.93, SD = 1293.99) than for functional products (M_display_ = 35,080.58, SD = 1327.28). The interaction effect of max fix pupil size between the region of interest and product type was significant (F (1,770) = 5.73, *p* = 0.013, *η*_p_^2^ = 0.008). In a further simple effects analysis of perfume, F (1,770) = 83.67, *p* < 0.001, *η_p_*^2^ = 0.098, the max fix pupil size was significantly larger for the virtual background area of interest (M_virtual background_ = 1513.08, SD = 31.44) than the patch area of interest (M_patch_ = 1105.89, SD = 31.52). In a simple effects analysis of displays, F (1,770) = 29.76, *p* < 0.001, *η*_p_^2^ = 0.037, the virtual background area of interest (M_virtual background_ = 1511.99, SD = 32.25) had a significantly larger max fix pupil size than the patch area of interest (M_patch_ = 1262.88, SD = 32.33).

The interaction effect of patch area of interest with unit fixation count on patch information was significant (F (2,770) = 5.09, *p* = 0.006, *η_p_*^2^ = 0.013). The main effect of patch region of interest was significant, F (2,770) = 5.781, *p* = 0.003, *η_p_*^2^ = 0.015, and the unit fixation count for pictures (M_picture_ = 0.261, SD = 0.02) was significantly smaller than that for the graphic–text combination (M_graphic-text combination_ = 0.330, SD = 0.02) and text (M_text_ = 0.334, SD = 0.02). The interaction effect of dwell time between the region of interest and patch information was significant (F (2,770) = 4.135, *p* = 0.016, *η_p_*^2^ = 0.011). The main effect of patch region of interest was significant, F(2,770) = 5.584, *p* = 0.004, *η_p_*^2^ = 0.014, and the dwell time for pictures (M_pictures_ = 17,405.08, SD = 1609.01) was significantly smaller than that for text (M_text_ = 23,911.10, SD = 1609.79) and the graphic–text combination (M_graphic-text combination_ = 24,068.29, SD = 1609.38), and the specific data are shown in Table 2.

**Figure 3:**
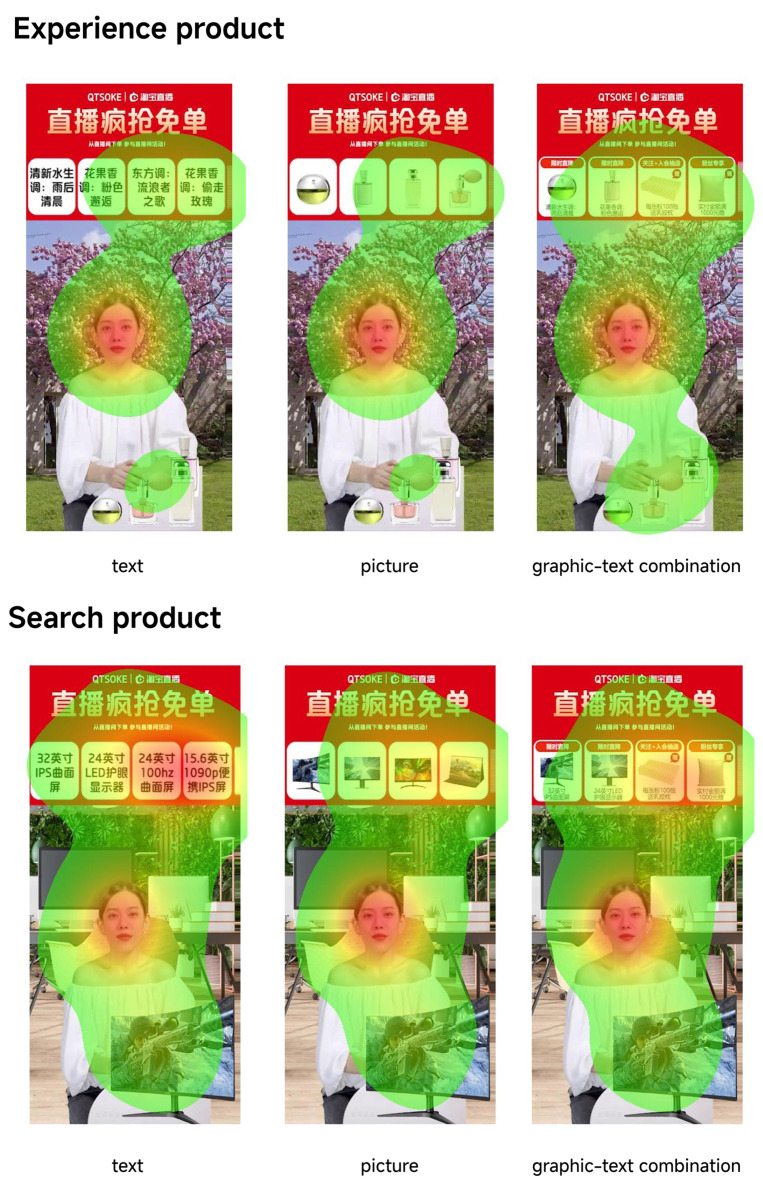
Eye movement heat map.

Heat maps show the distribution of eye movements on a display, providing intuitive behavioral information, and are a visualization method that can be effective in revealing the focus of visual attention. The visual heat map in Figure 3 was used to analyze the focus of attention, with dispersed eye movements indicating a longer search for a specific task, resulting in a longer fixation duration. Red-shaded (warm) areas indicate a longer fixation duration, while green-shaded (cool) areas indicate a shorter fixation duration. Thus, we can see the differences in the distribution of participants’ attention while watching different videos. For both product types, consumers gaze significantly longer at the patches and virtual backgrounds of the functional products than at the perfume products, while the opposite is true for the anchors, which is consistent with the characteristics of experience products and search products, i.e., consumers tend to judge the quality of the search products through figurative information such as words and pictures, whereas abstract verbal descriptions of the anchors are more likely to allow consumers to perceive the quality of the experience products (Kim, 2019; Sheng et al., 2021). For the patch information, it can be found from the display video that consumers’ fixation duration to the text patch is significantly longer than the combined graphic and text patch, while the picture patch has the shortest fixation duration, indicating that the textual information of the search product generates the highest level cognitive load, which is consistent with the results of the subjective questionnaires, and this partially supports the H1a hypothesis.

The analysis showed that revealed statistically significant main effects of both independent variables. For patch design information richness, the effect reached significance with F(12,708) = 5.174, *p* < 0.001, *η_p_*^2^ = 0.055. Similarly, product type showed significant influence F(6,355) = 4.999, *p* = 0.001, *η_p_*^2^ = 0.053. The post hoc test shows that the cognitive load of textual patches (M_text_ = 3.565) was significantly greater than that of graphic–text combination (M_graphic-text combination_ = 3.105) and graphic patches (M_graphic_ = 3.163), *p* = 0.015; *p* = 0.006. This partially supported H1a. Mental imagery was significantly lower for text patches (M_text_ = 4.033) than for picture (M_picture_ 4.346) and graphic–text combination patches (M_graphic-text combination_ = 4.578), supporting H1b. Text patches (M_text_ = 3.723) and picture patches (M_picture_ = 3.964) had significantly lower flow than graphic–text combination patches (M_graphic-text combination_ = 4.256), supporting H1c.

### 4.3. Model Analysis

#### 4.3.1. Mediation Effect Analysis

**Figure 4:**
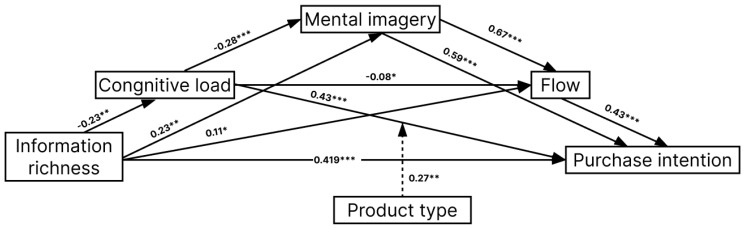
Structural model. Note: * *p* < 0.05; ** *p* < 0.01; *** *p* < 0.001.

We implemented multilevel mediation models using the lme4 and mediation packages in R. The analysis confirmed that all hypothesized paths were statistically significant, supporting the proposed mediation framework (see Figure 4). Patch design information richness, cognitive load, and mental imagery together accounted for 75.8% of the variance in flow, while cognitive load, mental imagery, and flow jointly explained 55.3% of the variance in purchase intention. These results suggest that cognitive load negatively affects customer experience and purchase decisions, whereas richer patch designs indirectly promote purchase intention by enhancing mental imagery and flow. Path coefficients and model estimates are summarized in Table 3.

#### 4.3.2. Moderated Mediation Analysis

To examine whether product type moderated the relationship between cognitive load and purchase intention, a linear mixed-effects model was conducted using the lmer Test package in R with standardized cognitive load. The results revealed a significant interaction between cognitive load and product type (CL × PT: b = −0.00003, SE = 0.000009, t(349.7) = −3.44, *p* < 0.001), indicating a moderation effect. Cognitive load positively predicted purchase intention overall (b = 0.00002, SE = 0.000006, t(338.5) = 2.45, *p* = 0.015), and the main effect of product type (PT2) was also significant (b = 0.676, SE = 0.353, t(268.6) = 1.92, *p* = 0.037).

**Figure 5:**
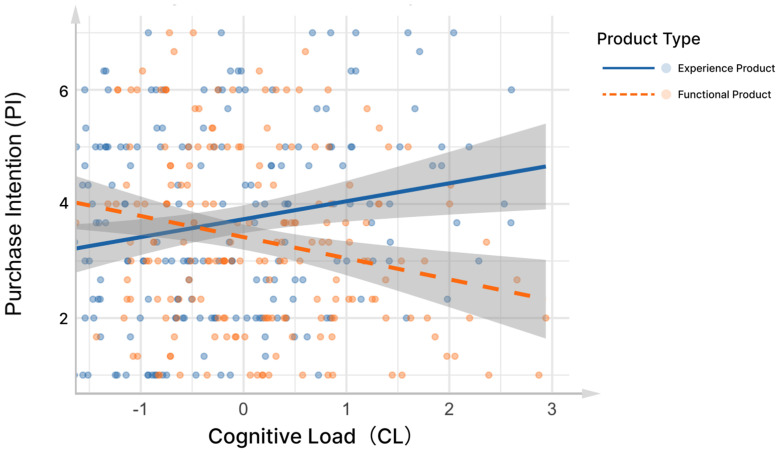
The effect of interaction between patch design and product type on purchase intention.

A simple slopes analysis (visualized in Figure 5) showed that the relationship between cognitive load and purchase intention was significantly positive for perfume products, but weakened or reversed for display products. These results support the hypothesis that product type moderates the indirect pathway from cognitive load to purchase intention within the proposed framework.

## 5. Discussion

This study uses a combination of experiments and questionnaires to explore the influence of visual elements in the live-streaming interface of online shops on users’ purchases. The results found that text and picture information in the live-streaming interface constitute different information richness, thus affecting customers’ cognitive load, mental imagery, and flow experience and that cognitive load, mental imagery, and flow experience play a mediating role in the influence of information richness of the live-streaming interface on customers’ purchase behavior.

The information richness of patch design in the live-streaming interface has an impact on the cognitive load of the customers, and the negative impact caused by text is the largest, and the use of a combination of graphics and text can significantly reduce the cognitive load. This indicates that, in the live-shopping interface, the conversion of abstract stimuli requires more brain work than the conversion of figurative stimuli (Ouwehand et al., 2021). The information richness of patch design in the live interface has a positive impact on the customers’ mental imagery, and the positive impact caused by text is the smallest, and the positive impact caused by using the combination of graphics and text is the largest. This indicates that the richer the information of the patch in the interface, the easier it is to trigger a sense of immersion or presence, providing users with a more convincing and attractive experience (L. Li et al., 2023). Pictures are more effective in the generation of mental imagery than text; thus, product images may generate richer mental imagery than product text (Jia et al., 2021). Although the text of a monitor summarizes the physical attributes and the text of a perfume summarizes the fragrance type, which is parametric information that is difficult to obtain in a product image, the color, texture, and other details displayed in a picture convey a sense of reality and touch ability, and therefore help customers imagine the actual quality of the product. (3) The information richness of patch design positively affects the flow, text has the least positive effect, and the combination of graphic and text patch design produces the most positive effect. This indicates that visual text is less likely to trigger a sense of presence in customers compared to pictures, making it more difficult for customers to immerse themselves and enter the state of flow.

While watching the live stream, the cognitive load of consumers negatively affects mental imagery and flow, which is consistent with our daily experience. The construction of visual images requires cognitive effort, and people’s cognitive resources are limited, and exhaustion limits their ability to process picture information; thus, cognitive load inhibits customers’ mental imagery. Mental imagery creates a sense of immersion and engagement, which makes it easier to enter the state of flow, while cognitive load clearly hinders this process.

While watching the live stream, mental imagery positively influences the flow and acts as an amplifier of positive emotions. It indicates that mental imagery has a non-negligible impact on changing emotional states, and in the field of live streaming, good visual effects and visual attraction enhance consumers’ emotions of pleasure, joy, and happiness, and mental imagery is stronger and easier to enter into the state of flow in the live-streaming interface rich in visual information (Abuhamdeh, 2020). For years, marketers have encouraged consumers to “use their imagination” because visualization has been shown to help increase purchase intentions (Babin & Burns, 1998). Mental imagery has an important role in shaping consumer behavior, especially in online environments where consumers rely on vivid visual cues to model experiences and increase engagement.

In addition, cognitive load negatively affects purchase intention and mental imagery and flow and positively affects purchase intention, which is also consistent with other hypotheses. Cognitive load negatively affects mental imagery and flow, which reduces customers’ purchase intention, while flow mediates the effect of mental imagery on customers’ purchase intention.

After adding product type moderation, it was found that cognitive load for perfume products significantly reduced purchase intention, while this process was not significant for displays. This may be due to the fact that purchases of experience products such as perfumes are driven by experiences, and therefore, negative experiences from cognitive load significantly reduce purchase intentions, whereas search products are less influenced by external factors as they are mainly driven by needs (Sheng et al., 2021).

Physiological data showed that the fixation count, first fixation time, dwell time, and max fix pupil size of live patches were significantly smaller than those of the background, which may be due to the fact that patches are much richer in information than the background area; thus, users tend to focus on the area with less cognitive load. This is consistent with Hadinejad et al. finding that adverts with less text attract more consumers’ attention (Hadinejad et al., 2020). However, in live-streaming commerce, complex interface designs may increase cognitive load, which can lead to attentional shifts from the advertised product to other areas of the screen, such as the background. This is especially true in information-rich designs, where overload may hinder users’ ability to engage with the key product message. Another reason could be that the anchor’s actions and words explaining the product attract more consumers than observing the product information. Among the functional products, consumers’ fixation counts and dwell times for picture patches were significantly smaller than other patches, and combined with the questionnaire data, it can be concluded that picture information produces the lowest cognitive load, and consumers’ mental imagery, flow, and purchase intention are the highest. Many eye-movement-related studies (Enders et al., 2021; Walter & Bex, 2022) have shown that regions with higher cognitive loads have more fixation counts and longer dwell times, and the opposite is true for regions with low cognitive loads, which supports our findings.

## 6. Conclusions

### 6.1. Theoretical Innovations

This study contributes theoretically to two main areas:

First, previous studies on live-streaming marketing generally focus on optimizing the achievement of marketing results, paying more attention to the “human” factors in the live-streaming process, such as internet celebrities and other online viewers, and ignoring the importance of “things” in live-streaming marketing. Live-streaming interface design can provide high quantities of information, give customers a vivid experience, and influence consumer behavior; thus, we should pay attention to it. Therefore, this study proposes a new perspective on the study of live-streaming marketing, focusing on the visual attractiveness of live-streaming interface design, exploring the impact of patch design on consumer purchase behavior, and enriching the research in the field of live-streaming marketing.

Second, this study is the first to focus on the changes in consumer behavior brought about by the flow theory in the field of live-streaming marketing. This study found that the information-rich live-streaming interface can produce a shopping experience in which interactivity, authenticity, and visualization coexist, which can help enhance consumers’ live-streaming purchase behavior. In this process, consumers’ cognitive load, mental imagery, and flow play an important mediating role. This finding enriches the research on the S-O-R theory and simultaneously bridges the research gap of mental emotions in live-streaming marketing.

Finally, this study also enriches related research in the field of consumers’ purchase intention. In the past, most of the studies on consumers’ purchase intention, especially on live purchase intention, focused on impulse consumption intention and herd consumption intention in live marketing, while there are not many studies exploring the factors influencing purchase intention from the perspective of graphic design. Starting from the design of the live-shopping interface, this study found for the first time that the information richness of patch design in the live interface can influence purchase intention. In a visual information-rich live-streaming interface, customers have a stronger sense of presence and stronger mental imagery and are more likely to enter the state of flow, thus increasing their purchase intention; however, cognitive load negatively affects the mental imagery and the flow, thus decreasing customers’ purchase intention. This finding provides a more in-depth exploration of the influencing factors of purchase intention in live streaming and enriches the relevant research on purchase intention.

### 6.2. Management Insights

This study provides valuable insights for marketing management:

First, this study clarifies how an information-rich live-streaming interface influences consumer psychology and purchase behavior. A carefully designed interface, combining interactive and visually engaging elements, enhances mental imagery and flow experiences, thereby boosting consumer engagement and purchase intentions. Businesses should carefully balance the information richness in their live-stream interfaces to engage consumers without causing cognitive overload.

Second, the findings highlight that consumer cognitive load, mental imagery, and flow experience significantly affect purchase decisions in live-streaming environments. Companies can strategically adjust visual and textual elements in advertisements to manage cognitive load effectively, enhance mental imagery, and foster flow experiences. Moreover, considering the moderating effect of product type, businesses should customize interface designs, i.e., rich visual content benefits experience products (e.g., perfumes), whereas detailed textual information is more suitable for search products (e.g., electronics).

Future research could further refine these strategies through A/B testing or case studies (Quin et al., 2023), providing practical recommendations to optimize live-streaming interfaces and enhance consumer engagement in a data-driven manner.

### 6.3. Shortcomings and Future Research Directions

Despite the experimental results supporting our hypotheses, some study limitations remain.

First, this study simulates live-streaming scenarios through material stimuli, which, while effective, may not fully reflect real-life purchasing experiences. Factors like real-time interaction with the host, promotions, and social influence were not adequately considered in this study (Ni & Ueichi, 2024). Future research should create more realistic marketing scenarios to improve the generalizability of the findings.

Second, while this study found varying effects of interface design on consumers’ purchase intentions, there are many other platform types and product categories that could influence results. Future studies should explore how different live-streaming platform types or more specific product categories impact purchase behavior.

Lastly, to deepen our understanding of consumer decision making, future research could incorporate emotional and physiological data (e.g., heart rate, facial expressions, etc.). This would provide insights into the real-time emotional and cognitive states of consumers, helping to refine how visual cues, emotional responses, and cognitive load influence purchase behavior in live-streaming environments.

## Figures and Tables

**Table 1:** Means and scales for each variable in different conditions.

Product Type	Information Richness	Cognitive Load		Mental Imagery		Flow		Purchase Intention	
		M	SD	M	SD	M	SD	M	SD
Experience Product	Text	3.55	1.31	4.07	1.22	3.18	1.23	2.85	1.45
	Picture	3.09	1.16	4.31	1.17	3.74	1.11	3.46	1.57
	Graphic–text combination	3.11	1.4	4.57	1.27	4.1	1.21	3.93	1.91
Functional Products	Text	3.07	1.26	3.8	1.19	3.18	1.21	2.74	1.47
	Picture	3.14	1.11	4.14	1.19	3.48	1.71	3.23	1.11
	Graphic–text combination	3.07	1.28	4.41	1.32	3.85	1.17	3.82	1.75

**Table 2:** Statistics of mean values of eye movement measurement data.

Product Type	Patch Design	Fixation Count	First Fixation Count	Dwell Time	Max Fix Pupil Size
Experience the product	Text	63.43 (43.70)	3217.54 (9486.00)	38,985.74 (29,493.97)	1315.67 (536.12)
	Picture	56.61 (34.73)	3059.09 (9852.51)	37,341.27 (28,709.41)	1316.49 (497.22)
	Graphic–text combination	65.07 (40.88)	7019.13 (19,696.37)	37,939.21 (27,942.02)	1297.71 (513.70)
Search product	Text	59.07 (45.41)	3517.82 (11,494.47)	28,898.37 (18,699.69)	1438.20 (465.04)
	Picture	47.77 (29.80)	2851.89 (15,001.64)	25,673.56 (17,646.88)	1339.73 (428.01)
	Graphic–text combination	59.09 (42.99)	2749.58 (7857.85)	30,291.57 (19,766.75)	1383.81 (439.79)

**Table 3:** Model analysis of the effect of patch design information richness on purchase intention.

Hypothetical Path	Non-Standardized Coefficients			*p*	R^2^
	Corf.	Standard Error	β		
H1: IR → PI	0.419	0.173	0.812	<0.001	0.567
H2a: IR → CL	−0.23	0.273	−0.15	0.01	0.338
H2b: IR → MI	0.23	0.09	0.15	0.07	0.505
H3a: CL → MI	−0.28	0.07	−0.17	0.001	
H2c: IR → FLOW	0.11	0.05	0.08	0.032	0.758
H3b: CL → FLOW	−0.08	0.17	−0.09	0.019	
H4: MI → FLOW	0.67	0.21	0.64	<0.001	
H5a: CL → PI	0.43	0.12	0.18	<0.001	0.553
H5b: MI → PI	0.59	0.19	0.41	<0.001	
H5c: FLOW → PI	0.43	0.13	0.22	<0.001	

Note: IR = information richness; CL = cognitive load; MI = mental imagery; PI = purchase intention.

## Data Availability

The original contributions presented in the study are included in the article. Further inquiries can be directed to the corresponding author.
